# Obesity Facts and Their Influence on Renal Function Across the Life Span

**DOI:** 10.3389/fmed.2021.704409

**Published:** 2021-11-12

**Authors:** Vera H. Koch

**Affiliations:** Pediatric Nephrology Unit, Department of Pediatrics, Instituto da Criança e do Adolescente do Hospital das Clínicas da Faculdade de Medicina da Universidade de São Paulo, São Paulo, Brazil

**Keywords:** obesity, glomerular filtration rate, creatinine, cystatin C, pediatric, adult

## Abstract

Obesity is a chronic disease, with a rapidly increasing prevalence worldwide. Body mass index (BMI) provides the most useful population-level measure of overweight and obesity. For adults, overweight is defined as a BMI (Kg/m^2^) ≥ 25, and obesity as a BMI ≥ 30, for non-Asians and ≥ 27.5 for Asians. Abdominal obesity can be defined as a waist circumference equal to or higher than 102 cm for men and ≥88 cm for women. The definition of children and adolescents BMI changes with age and sex. Obesity may be exogenous or endogenous obesity, the latter is multifactorial and predominantly manifested during childhood. Presently, overweight and obesity are linked to more deaths worldwide than underweight. The total kidney glomerular filtration rate (GFR) is determined by the sum of nephrons and the GFR within each nephron or single nephron GFR. In clinical practice, GFR is more frequently calculated by GFR estimating equations based upon the plasma levels of creatinine, cystatin C, or both. The measured value of plasma creatinine is strongly influenced by non-GFR factors, by its tubular and gastrointestinal secretion, and by the problems associated with the lack of standardization of creatinine's laboratory assay discrediting it as an ideal GFR biomarker. Unlike creatinine, cystatin C plasma levels are mainly determined by GFR. Obesity may affect the kidney, *via* development of systemic arterial hypertension and/or diabetes mellitus, or directly, by ectopic accumulation of adipose tissue in the kidney. As obesity is a clinical condition associated with altered body composition, creatinine may not be the ideal biomarker for GFR measurement in obese individuals.

## Introduction

The global prevalence of obesity has nearly tripled between 1975 and 2016. Approximately 13% of the world's adult population (11% of men and 15% of women) could be classified as obese in 2016. In the same period, the prevalence of overweight and obesity among children and adolescents, aged 5–19, of both sexes, has risen from 4% in 1975 to over 18% in 2016^1^.

Obesity is a chronic disease, which results from long-term positive energy balance with development of excessive body fat mass. It leads to structural and functional abnormalities potentially associated with an elevated to premature mortality risk (1). Body mass index **(**BMI) provides the most useful, although rough, population-level measure of overweight and obesity but as BMI cannot differentiate between lean and fat body mass, it may not correspond to the same degree of fatness in different individuals ^1^.

## The Definition of Overweight and Obesity Across the Lifespan

For adults, overweight is defined as a BMI ≥ 25 Kg/m^2^, and obesity as a BMI ≥ 30 Kg/m^2^, for non-Asians and ≥27.5 Kg/m^2^ for Asians and abdominal obesity can be defined as a waist circumference ≥102 cm for men and ≥88 cm for women (2)^1^.

In children and adolescents, growth, and maturation lead to changes of BMI with age and sex. There are at least three different definition proposals for child and adolescent overweight and obesity, which have been issued by the WHO, the Center for Disease Control and Prevention CDC) and by the International Obesity Task Force (IOTF). The WHO criteria recommend utilization of BMI for adolescents and weight- for- height/ length *Z*-score for children (3). The Center for Disease Control and Prevention (CDC) has derived sets of percentiles of age and sex- specific BMI for children and adolescents aged 2–20 years, in the United States (4). The International Obesity Task Force (IOTF) obtained data on body mass index (weight/height) from six large cross-sectional surveys on growth from Brazil, Great Britain, Hong Kong, the Netherlands, Singapore, and the US, including 97,876 males and 94,851 females from birth to 25 years of age. For each of the surveys, centile curves were drawn passing through the established cut-off points of 25 and 30 kg/m^2^ for adult weight and obesity, at age 18 years. The resulting curves were averaged to provide age- and sex- specific cut-off points from 2 to 18 years (5). Despite a consensus between the two most frequently used criteria, CDC and IOTF, their discrepancies about age, gender, and country are noticeable and render the estimated prevalence rates of overweight and obesity non-comparable worldwide (6).

For children >2 years of age, using the CDC criteria, overweight may be defined as BMI ≥85th percentile but <95th percentile for age and sex, obesity as BMI ≥95th percentile, and extreme obesity if the BMI is ≥120% of the 95th percentile or ≥35 kg/m^2^ (7). For children <2 years of age, obesity may be defined as the sex-specific weight for recumbent length ≥97.7th percentile on the World Health Organization (WHO) charts (7). A new classification system recognizes BMI ≥95th percentile as class I obesity, BMI ≥ 120% of the 95th percentile as class II obesity, and BMI ≥ 140% of the 95th percentile as class III obesity. Class II and III obesity are strongly associated with greater cardiovascular and metabolic risk (8).

Table 1 presents a summary of the cut -off values used to define overweight and obesity in the pediatric age range and in adulthood.

**Table 1:** Cut-off values used to define overweight and obesity in childhood and adulthood.

**Overweight**	**Obesity**
**CHILDREN AND ADOLESCENTS**	
**CDC Children 2–20 years of age**	
BMI ≥85th percentile but <95th percentile for age and sex	BMI ≥95th percentile
**WHO children <5 years of age**	
BMI or Weight for length/ height > +2SD WHO Child Growth Standards median	Obese: BMI or Weight for length/ height > +3SD WHO Child Growth Standards median
**WHO 5–19 years of age**	
BMI > +1SD WHO Growth Reference median;	BMI > +2 SD WHO Growth Reference median;
**IOTH 2–18 years of age**	
Age- and sex- specific cut-off points to correspond to BMI cut-offs of 25 kg/m^2^ at 18 years of age	Age- and sex- specific cut-off points to correspond to BMI cut-offs of 30 kg/m^2^ at 18 years of age
**ADULTS**	
BMI ≥ 25 Kg/m^2^	BMI ≥30 Kg/m^2^ for non- Asians and ≥ 27.5 Kg/m^2^ for Asians

*Source: Modified from references (1, 2, 7, 9)^1^*.

*CDC, Centers for Disease Control and Prevention; IOTF, International Obesity Task Force; BMI, Body Mass Index; WHO, World Health Organization*.

Waist circumference (WC), waist-to-hip ratio and waist-to-height ratio (WHtR) reference percentiles are used to define abdominal obesity in children and adolescents, these indices may vary according to ethnicity (10–13). Observations from pediatric and adult studies suggest WHtR and WC to be independent and more significant predictors of cardiometabolic outcomes than BMI, Mean threshold values for WHtR, covering all cardiometabolic outcomes, from studies in fourteen different countries and including Caucasian, Asian and Central American subjects, were 0.50 for men and 0.50 for women. The AUROC analyses indicate that WHtR may be a more useful global clinical screening tool than WC, with a weighted mean threshold value of 0.5, or in simpler terms, for public health purposes, the “waist circumference should be kept to less than half the individual's height” (14).

## Obesity as A Multifactorial Entity

### The Etiology of Obesity May Be Endogenous or Exogenous

The development of exogenous or acquired obesity requires chronic overconsumption, leading to storage of excess caloric intake as lipid in fat cells, without behavioral or metabolic compensation (15). In healthy individuals, 70–95% of insulin-mediated glucose disposal is undertaken by skeletal muscle-cells. The functional compromise of skeletal muscle-cell metabolic-flux and concomitant insulin sensitivity, which are the main determinants of metabolic control, lead to increased availability of glucose or other serum energy substrates for adipogenesis. Of note, the major modifiable determinant of skeletal muscle cell insulin sensitivity is physical activity, extremely low levels of physical activity decreases both insulin sensitivity and metabolic control (15).

Over the past few generations, physical activity and fitness levels declined in both children and adults (16). When an individual's physical activity falls below the “metabolic tipping point,” defined as the minimum amount of physical activity and associated metabolic flux, necessary to avoid positive energy- balance, acquired obesity develops (15).

Various monogenic, genetic syndromic, and endocrine conditions may lead to endogenous obesity, which is predominantly manifested during childhood. The assessment of childhood endogenous obesity should be based on a very thorough history and physical examination directed at etiological elucidation and diagnosis of the associated metabolic, cardiovascular, respiratory, gastrointestinal, orthopedic, and psychological complications (9). Endocrine causes, although rare (2–3% of all referrals for pediatric obesity evaluation) should be carefully evaluated so that specific treatment can be provided (16). Children born small or large- for- gestational age, infants of diabetic mothers and those with rapid or excessive catch-up growth in the first few years of life are frequently conditioned by metabolic programming, which exacerbates the problem of obesity associated with lifestyle and dietary factors (17). Table 2 lists the main etiologies associated with endogenous and exogenous obesity.

**Table 2:** Main etiologies associated with endogenous and exogenous obesity.

**EXOGENOUS OBESITY**	
Chronic overconsumption	Storage of excess caloric intake as lipid in fat cells, without behavioral or metabolic compensation
Medications	Glucocorticoids, tricyclic antidepressants, risperidone
Adverse perinatal metabolic programming	Children born small or large- for- gestational age
**ENDOGENOUS OBESITY**	
*Monogenic diseases*	
Recessive defects in genes	
Leptin (LEP)	Extreme hyperphagia, frequent infections, hypogonadotropic hypogonadism, hypothyroidism
Leptin receptor (LEPR)	Same as LEP
Pro-opiomelanocortin (POMC)	Hyperphagia, cholestatic jaundice or adrenal crisis due to ACTH deficiency, pale skin, and red hair in Caucasians
Proprotein convertase subtilisin/kexin type 1 (PCSK1)	Small bowel enteropathy, hypoglycemia, hypothyroidism, ACTH deficiency, diabetes insipidus
Dominant defects in genes	
Melanocortin 4 receptor (MC4R),	Hyperphagia, accelerated linear growth, disproportionate hyperinsulinemia, low/normal blood pressure
Src homology 2 B adapter protein 1 (SH2B1)	Hyperphagia, disproportionate hyperinsulinemia, early speech, and language delay with frequent good resolution, behavioral problems
Kinase suppressor of Ras 2 (KSR2)	Mild hyperphagia and reduced basal metabolic rate, insulin resistance acanthosis nigricans is a frequent feature, irregular menses, early development of type 2 diabetes mellitus
Syndromic conditions	
Prader-Willi syndrome	Hypotonia and failure to thrive in infancy with subsequent weight gain, short stature, hyperphagia, hypogonadotropic hypogonadism, developmental delay
Albright hereditary osteodystrophy	Skeletal defects, short stature may be present impaired olfaction, developmental delay and hormone resistance (e.g., parathyroid hormone) if mutation is maternally derived
Bardet-Biedl syndrome	Dysmorphic extremities (syndactyly/brachydactyly/polydactyly), retinal dystrophy or pigmentary retinopathy, renal abnormalities, hypogonadism, developmental delay
Alstrom syndrome	Retinal dystrophy; extreme insulin resistance; deafness; dilated cardiomyopathy; progressive pulmonary, hepatic, and renal dysfunctions
Endocrinopathies	
Hypothyroidism Cushing syndrome, Hypothalamic obesity, Growth hormone deficiency Persistent hyperinsulinism	

*Source: modified from references (7, 9)*.

Presently, overweight and obesity are linked to more deaths worldwide than underweight^1^. An analytical study carried out on 10,625,411 subjects in Asia, Australia and New Zealand, Europe, and North America, from 239 prospective studies, evaluated mortality risk, paired by sex and age, compared to BMI 22.5–25.0. The study confirmed the associations of both overweight and obesity, with increased mortality from any cause, within the four continents (18).

Prediabetes /type 2 diabetes mellitus; dyslipidemia; prehypertension / hypertension; sleep apnea; non-alcoholic fatty liver disease; proteinuria and focal segmental glomerulosclerosis; early subclinical atherosclerosis; cardiovascular disease (CVD), hyperandrogenemia/ polycystic ovary syndrome, and orthopedic problems are among the multiple clinical comorbidities associated with pediatric overweight and obesity. Obesity severity is associated with higher cardiometabolic risk factors and premature mortality in adulthood (7). Of note, the risks of CVD outcomes among obese children and adolescents who became non-obese by adulthood are similar to those who were never obese (19).

## Glomerular Filtration Rate Determination

Urine is the product of ultrafiltration of blood plasma through a multilayered structure known as glomerular filter, which is composed, by the fenestrated endothelium, glomerular basement membrane, and glomerular epithelium or podocyte layer (20). The glomerular filtration rate (GFR) can be expressed as the volume of plasma that can be completely cleared of a substance in a time unit and is usually expressed as milliliters per minute (ml/min). To facilitate comparison of GFR among children and adults, body surface standardization to a body surface area of 1.73m^2^ is used, and GFR is expressed as ml/min/1.73m^2^. The evaluation of GFR in children, should also take into consideration the postnatal kidney maturation which leads to an increase in GFR from low gestational age- dependent levels at birth to the adult level of 120 mL/min/1.73 m^2^ which is reached at ~2 years of age (21).

The total kidney glomerular filtration rate (GFR) is determined by the sum of nephrons and the GFR within each nephron or single nephron GFR A decline in kidney function can be secondary to a decrease in SNGFR because of systemic conditions such as hypoperfusion, and/or by a reduction in nephron number (21).

The simplified functional pore model is the most frequently utilized model to describe the filtration process. The sieving coefficient, which is the ratio of the concentration of each molecule in the urine and in plasma, characterizes the filtration of molecules of different sizes (22). As the sieving coefficients for small molecules <1 kDa are 1, the substances belonging to this group that, contrary to creatinine, do not undergo tubular reabsorption or secretion, such as 51Cr- EDTA (0.34 kDa), iohexol (0.82 kDa), 125I-iothalamate (0.64 kDa) are frequently used to measure GFR. Inulin, an inactive, uncharged 5.2 kDa polymer of fructose, presents many of the characteristics of an ideal marker and its clearance is to date the gold standard for this measurement (21–23).

The plasma disappearance or the urinary excretion of an intravenous injection of a non-radioactive low molecular weight substance such as Iohexol, is particularly suitable to evaluate GFR in children and fertile women (23). However, in the interest of patient comfort, time, and cost, in clinical practice, GFR is generally calculated by *GFR estimating equations* based upon the plasma levels of creatinine (SCr), cystatin C (sCysC) or both. The measured value of plasma creatinine is strongly influenced by non -GFR factors such as muscle mass, growth, diet, and illness, by its tubular and gastrointestinal secretion and by the problems associated with the lack of standardization of its laboratory assay. As a consequence, SCr is considered a suboptimal GFR biomarker (21). Given the limitations of SCr, creatinine-based formulas include surrogates for muscle mass, such as height, weight, and gender, which is especially useful in children and adolescents, as in the pediatric age range, the level of SCr corresponding to normal kidney function /GFR, varies with age (21).

The first pediatric GFR formulas were derived independently by Schwartz and Counahan-Barratt in the mid-1970's. Although both equations were developed in children with chronic renal disease, and use height as a surrogate for growth, they present different proportionality constants reflecting the relationship between urinary creatinine excretion and body size. The original Schwartz formula yields a constant at 0.55, has been derived using a modified Jaffe creatinine assay and inulin clearance as the reference standard (24, 25). The Counahan Barratt formula, uses the plasma clearance of intravenously administered 51- chromium edetic acid (5"Cr-EDTA) as reference standard and yields a proportionality constant of 0.43. It has been derived using creatinine determination by Jaffe reaction, after adsorption by an ion-exchange resin to remove non-creatinine chromogens (26).

The Jaffe assay, an alkaline picrate colorimetric reaction, is affected by the presence of interfering, non-creatinine chromogens, which may falsely elevate SCr by up to 20%, especially at lower levels of creatinine, as observed in the 1st year of life (25). An enzymatic assay for creatinine determination has been developed resulting in 20–30% lower creatinine levels and which are more consistent with accurate HPLC- derived creatinine values (27, 28). To enable the use of the less expensive Jaffe method for creatinine determination in lower resource areas, a correction for a constant bias as compared to the Isotope Dilution Mass Spectrometry (IDMS) reference method has been introduced, accounting for the contribution of serum protein to Jaffe's creatinine, yielding a creatinine value closer to that obtained using an enzymatic creatinine assay (29). Use of an IDMS-traceable creatinine value with the original Schwartz equation will overestimate GFR by 20–40% (21).

More recently, more precise creatinine-based GFR estimation have yielded full - age -spectrum (FAS) equations, such as the Lund–Malmo revised (LMR) equation LMR18 and the new European Kidney Function Consortium (EKFC) equation, valid for both children and adults (30, 31). The development of FAS equations provide a seamless definition of normality values of GFR progression from adolescence to adulthood and from adulthood to old age. Their utilization may yield age- specific GFR values and an age -adapted definition of chronic renal disease, in consonance with morbidity and mortality data, which may result in a much lower global chronic kidney disease prevalence particularly for elderly individuals (32). In young adults, 18–26 years of age, eGFR may be underestimated with the pediatric CKiD formula (28) and overestimated by the adult CKD- EPI equation (33). Of note, averaging results from the pediatric and adult formulas yields an eGFR result which is close to the one obtained by an iohexol GFR determination (21).

Serum cystatin C (sCys-C), a 13 kDa cationic cysteine protease inhibitor is produced, by all nucleated cells, at a constant rate. sCys-C is freely filtered by the glomerulus; it does not undergo tubular secretion and is reabsorbed and metabolized by the proximal tubule epithelial cells, high concentrations of CysC can be demonstrated in the urine from patients with renal tubular dysfunction, due to defective reabsorption (34, 35). Unlike creatinine, sCys-c plasma levels are mainly determined by GFR, without significant influence of age, gender, and muscle mass. s-Cys-c does not seem to be trans-placentally exchanged, In children, sCys-c highest concentration occurs on the 1st day of life. s-CysC levels are higher in preterm than full-term infants, rapidly decreasing during the next months of life. s CysC levels tends to stabilize by ~1.5–2 years of age (21, 36) or 3 years of age (37). sCys-C levels may be elevated by systemic inflammation, therapeutic use of corticosteroids and by hyperthyroid states, while sCys-C reduced levels may accompany hypothyroid conditions (35). These characteristics suggest. sCys-C as an ideal biomarker of kidney function in children and, in selected clinical situations with altered body composition or reduced muscle mass, such as malnutrition and neuromuscular disease (34).

Reference values for sCys-C obtained in a selected population of healthy individuals, by nephelometry were 0.75+/−0.089 mg/l for children aged 4–19 years, 0.74+/−0.100 mg/l for males, and 0.65+/−0.085 mg/l for females (aged 20–59 years), and 0.83+/-0.103 mg/l for older individuals (> or =60 years) (38).

Of note, sCysC independence of muscle mass enables the use of the same s- Cys C-based GFR estimating equation for children and adults (39). The Caucasian-Asian-Pediatric-Adult (CAPA) equation can be utilized for all individuals above 1 year of age and differently from creatinine-based equations, does not require inclusion of sex and “race” variables, to compensate for differences in muscle mass between individuals (40).

Interestingly, GFR estimates based on sCys-C equations do not offer precision advantages in comparison with creatinine-based estimates, probably because unmeasured and unidentified non-GFR determinants of cystatin C are as important as those of creatinine. Equations that combine creatinine and sCys-C offer, in general, the most accurate estimate of GFR across the range of GFRs and in subgroups based on demographic and clinical characteristics. This finding may be explained because although errors due to non-GFR determinants of creatinine and sCys-C, are still present in combined equations, their effect is minimized in an equation that uses both markers simultaneously (41). GFR estimating equations combining sCys-C and creatinine have been generated for children (42–44) and adults (41, 45).

The Chronic Kidney Disease in Children study, is a cohort of ~600 children with chronic kidney disease (CKD) in the United States and Canada. The study focuses on the identification of risk factors for GFR decline and renal disease progression, cardiovascular morbidity, growth failure, and neurocognitive impairment. Utilizing the methodology of iohexol plasma disappearance (iGFR) as a basis for the development of GFR estimating equations, the CKiD study has created the updated version of the bedside creatinine-based GFR equation (21, 28), which is recommended by the National Kidney Disease Education Program for use when creatinine is measured using IDMS methods., as well as, a bedside Cystatin C based GFR equation (21) and a combined GFR equation which include serum creatinine, blood urea nitrogen, height, gender, and cystatin C (21, 44). The study initially chose to measure sCys-C by an immunoturbidimetric method, which provided technically inferior results, motivating the change to immunonephelometry. The reciprocal of immunonephelometric cystatin C correlated as well with GFR as height/ serum creatinine ratio (both 0.88). The resulting combined equation, shown among others, in Table 3, is highly accurate and precise, providing good results in children with CKD in a GFR range from 15 to 75 ml/min per 1.73 m^2^. It is being utilized to estimate GFR in the follow up of the CKiD cohort in the clinical visits when iohexol is not administered. eGFR equations perform best when applied with the same laboratory methodology and patient population characteristics that were used in its development. The new Schwartz equations were developed using data from pediatric patients with chronic kidney disease and an isotope dilution mass spectrometry (IDMS)-traceable enzymatic creatinine method. Studies to establish its applicability to children of normal growth and muscle mass, and higher GFR are needed (44).

**Table 3:** Creatinine- based, cystatin based and combined creatinine/cystatin GFR Estimating Equations (ml/min/1.73 m^2^) that have been developed for use in pediatric individuals.

**CREATININE—BASED GFR ESTIMATING EQUATIONS**			
Schwartz “bedside (original- 1976) in mL/min/1.73 m^2^ (24, 25)	eGFR = k × L (cm)/PCr (mg/dL) where k ~ 0.33 (preterm infant), k ~ 0.45 (full term), k ~ 0.55 (children and adolescent females), k ~ 0.7 (adolescent males)		
Counahan-Barrat in mL/min/1.73 m^2^ (26)	eGFR = 0.43 × L (cm)/PCr (mg/dL)		
Updated Schwartz (CKiDCr in mL/min/1.73 m^2^) (28)	eGFR = 0.413 × L (cm)/PCr (mg/dL)		
Pottel Belgium/Pottel Lyon Equation in mL/min/1.73 m^2^ (46, 47)	eGFR = 107.3 /(SCr/Q) where Q is median serum creatinine concentration of the average healthy individual in that population. based on age and sex (for Q values up to 20 years of age see Table 5)		
**New Pottel equation**			
	**Age**	**SCr/Q**	**Equation**
New Pottel et al. (31)	2–40 yr	<1	**107.3 × (SCr/Q)** ^**−0.322**^
		≥1	**107.3 × (SCr/Q)** ^**−1.132**^
	> 40 yr	<1	**107.3 x (SCr/Q)**^**−0.322**^ **× 0.990**^**(Age−40)**^
		≥1	**107.3 × (SCr/Q)**^**−1.132**^ **× 0.990**^**(Age−40)**^
**CYSTATIN C - BASED GFR ESTIMATING EQUATIONS**			
CKiDCys-C (Schwartz “bedside” cystatin C) in mL/min/1.73 m^2^ (21)	eGFR = 70.69 × [cystatin C (mg/L)]^−0.931^		
CAPA Equation (>1 year of age) in in mL/min/1.73 m^2^ (40)	eGFR=130 x cystatin C^−1.069^ × age^−0.117^-7		
Larsson (ml/min) (48)	eGFR = 77.24 × (Cys^−1.2623^)		
Le Bricon (in mL/min/1.73 m^2^) (49)	eGFR= (78/Cys) + 4		
Filler (in mL/min/1.73 m^2^) (50)	Log(eGFR) = 1.962 + [1.123 × log(1/cystatin)]		
Zappitelli ((ml/min pe/ 1.73 m^2^) (43)	eGFR = 75.94 × Cys^−1.17^		
**COMBINED CREATININE/CYSTATIN C GFR ESTIMATING EQUATIONS**			
CKiD (44)	eGFR = 39.8*[ht(m)/Scr]^0.456^[1.8/cysC]^0.418^[30/BUN] ^0.079^1.076^gender^[ht(m)/1.4]^0.179^ gender 1 male; 0 female		
Zappitelli combined creatinine—cystatin C (ml/min / 1.73 m^2^) (43)	eGFR = (507.76 × e^0.003 × height)^/(Cys ^0.635^ × Cr^0.547^) ×1.165 if renal transplant		
Bouvet (in ml/min) (42)	eGFR = 63.2 × (Cr/96)^−0.35^ × (Cys/1.2)^−0.56^ × (weight/45)^0.30^ × (age/14)^0.40^		

*eGFR, estimated GFR L ~ length/height in cm; PCr/SCr serum creatinine in mg/dL, Cystatin C in mg/L*.

Of note, the arithmetic mean of the results of a cystatin C based GFR estimating equation and a creatinine-based estimating equation has been proven to perform as well as, or even better, for adults and children, than complex combined equations (51–55).

The most frequently used GFR determination equations are the updated Schwartz creatinine formula in children (CKiD) (28) and the CKD-EPI Creatinine Equation in adults (41). Tables 3, 4 present respectively, a list of some of the pediatric and adult GFR estimating equations. Table 3 includes the Pottel height- independent GFR estimation equation, based on levels of enzymatic / isotope dilution mass spectrometry (IDMS) –equivalent assay for serum creatinine, in mg/dL (46, 47), whose performance is comparable to the height-dependent (Schwartz) equations in the identification of renal dysfunction (GFR <75 mL/min/1.73 m^2^) in children (46). Table 4 focuses on the CKD-EPI (56) and Modification of Diet in Renal Disease (MDRD) based on serum creatinine values standardized to isotope dilution mass spectroscopy (IDMS), devised for adult individuals. Of note the MDRD Study equation despite its development in individuals with CKD, has gained widespread use. Estimated GFR using this equation is reported by most clinical laboratories when measurement of serum creatinine is ordered, which is inadequate because although the MDRD equation performs well in patients with CKD, it systematically underestimates measured GFR at higher levels such as potential kidney donors, young people with type 1 diabetes and patients with substantially reduced muscle mass (45). Table 5 presents median serum creatinine concentrations (Q) for pediatric and adult individuals to be used in the height independent GFR estimating equations developed by Pottel et al. (46, 57, 58).

Table 4Creatinine Equation (CKD-EPI 2009), Cystatin C Equation (CKD-EPI 2012), and Creatinine–Cystatin C Equation (CKD-EPI 2012) for Estimating GFR, Expressed for Specified Sex, Serum Creatinine Level, and Serum Cystatin C Level (41) and MDRD equation (56) based on SCr values standardized to isotope dilution mass spectroscopy (IDMS) developed for use in adult individuals.

**Table:** 

	**Serum Creatinine (SCr) (mg/dL)**		
**CKD-EPI creatinine equation**	Female	≤ 0.7	144 × (Scr/0.7)^−0.329^ × 0.993^Age^ [ ×1.159 if African American]
	Female	>0.7	144 × (Scr/0.7)^−1.209^ × 0.993^Age^ [ ×1.159 if African American]
	Male	≤ 0.9	141 × (Scr/0.9)^−0.411^ × 0.993^Age^ [ ×1.159 if African American]
	Male	>0.9	141 × (Scr/0.9)^−1.209^ × 0.993^Age^ [ ×1.159 if African American]
	**Serum Cystatin C (SCys) (mg/L)**		
**CKD-EPI Cystatin C equation**	Female or male	* ≤ 0.8*	133 × (Scys/0.8)^−0.499^ × 0.996^Age^ [ × 0.932 if female]
	Female or Male	>0.8	133 × (Scys/0.8)^−1.328^ × 0.996^Age^ [ × 0.932 if female]

**Table:** 

		**SCr**	**SCys**	
**CKD-EPI creatinine– cystatin C equation**	Female	≤ 0.7	≤ 0.8 >0.8	130 × (Scr/0.7)^−0.248^ × (Scys/0.8)^−0.375^ × 0.995^Age^ [ ×1.08 if African American]
				130 × (Scr/0.7)^−0.248^ × (Scys/0.8)^−0.711^ × 0.995^Age^ [ ×1.08 if African American]
	Female	>0.7	≤ 0.8	130 × (Scr/0.7)^−0.601^ × (Scys/0.8)^−0.375^ × 0.995^Age^ [ ×1.08 if African American]
			>0.8	130 × (Scr/0.7)^−0.601^ × (Scys/0.8)^−0.711^ × 0.995^Age^ [ ×1.08 if African American]
	Male	≤ 0.9	≤ 0.8 >0.8	135 × (Scr/0.9)^−0.207^ × (Scys/0.8^)−0.375^ × 0.995^Age^ [ ×1.08 if African American]
				135 × (Scr/0.9)^−0.207^ × (Scys/0.8^)−0.711^ × 0.995^Age^ [ ×1.08 if African American]
	Male	>0.9	≤ 0.8 >0.8	135 × (Scr/0.9^)−0.601^ × (Scys/0.8^)−0.375^ × 0.995^Age^ [ ×1.08 if African American]
				135 × (Scr/0.9^)−0.601^ × (Scys/0.8)^−0.711^ × 0.995^Age^ [ ×1.08 if African American]

**Table:** 

MDRD Study equation	Serum creatinine values standardized to isotope dilution mass spectroscopy (IDMS)	GFR = GFR (mL/min/1.73 m^2^) = 175 × (S_cr_)^−1.154^ × (Age)^−0.203^ × (0.742 if female) × (1.212 if African American)

**Table 5:** Q-values [=median serum creatinine in μmol/L (mg/dL)], according to age or height, for height -independent full age spectrum SCr equation by Pottel et al. (46, 57, 58).

**Age, years**	**Height, cm**	**Qb, μmol/L (mg/dL)**
**BOYS AND GIRLS**		
1	75.0	23 (0.26)
2	87.0	26 (0.29)
3	95.5	27 (0.31)
4	102.5	30 (0.34)
5	110.0	34 (0.38)
6	116.7	36 (0.41)
7	123.5	39 (0.44)
8	129.5	41 (0.46)
9	135.0	43 (0.49)
10	140.0	45 (0.51)
11	146.0	47 (0.53)
12	152.5	50 (0.57)
13	159.0	52 (0.59)
14	165.0	54 (0.61)
**MALE ADOLESCENTS**		
15	172.0	64 (0.72)
16	176.0	69 (0.78)
17	178.0	72 (0.82)
18	179.0	75 (0.85)
19	180.0	78 (0.88)
**MALE ADULTS**		
≥20	≥181.5	80 (0.90)
**FEMALE ADOLESCENTS**		
15	164.5	57 (0.64)
16	166.0	59 (0.67)
17	166.5	61 (0.69)
18	167.0	61 (0.69)
19	167.5	62 (0.70)
**FEMALE ADULTS**		
≥20	≥168.0	62 (0.70)

*Obs: Height is the median height of a child or adolescent at the specified age (Belgian growth curves)*.

In neonates sCys-C is a better biomarker of GFR than creatinine. During pregnancy, the placenta is responsible for fetal creatinine equilibration, at birth the neonate SCr reflects the mother's SCr levels, as the neonate's kidney takes control, there is an initial increase in the neonate's SCr followed by a gradual fall along the first 2 years of life when the kidneys reach full maturation. sCys-C levels at birth are elevated on account of the physiologically lower GFR of the neonate. Kidney maturation leads to increases in GFR causing, sCys-C levels to fall reaching a plateau after 18 months of age (21, 36, 37). A recent multicentric worldwide systematic review, from 10 countries across four continents, described sCysC progression, in 1,468 babies born preterm, along the 1st month of life, infants born at 24–28 weeks of gestational age (GA), presented s- CysC values ranging from 1.44 to 1.90 mg/L, on day 1, to 1.36 to 2.02 between 4 and 30 days of life, while in preterm infants born after 34 weeks of gestational age, sCys C values ranged from 1.22 to 1.96 mg/L, on day 1, to 1.22 to 1.82 between 4 and 30 days of life (59). Mean sCys C values cystatin C, usually fall to a mean concentration of 0.80 mg/liter by the 1st year of life (34).

## Obesity and the Kidney

The renal effects of obesity can be indirect, *via* development of systemic arterial hypertension and / or diabetes mellitus, or direct, by adipose tissue hypertrophy and its ectopic accumulation in the kidney. Ectopic renal accumulation of lipids lead to multiple functional and structural changes, which although not fully understood, may lead to glomerular hypertension, increased glomerular permeability, hyperfiltration, glomerulomegaly, albuminuria, and in some cases, focal and segmental glomerulosclerosis (FSGS) characterizing the obesity related glomerulopathy (60–63).

The Chronic Kidney Disease Prognosis Consortium (CKD-PC) study evaluated the association between various measures of adiposity with eGFR deterioration and mortality from all causes. The included participants were 18 years and older, eGFR, was calculated utilizing the CKD-EPI equation. BMI data were collected from 39 general population, six high CVD risk, and 18 CKD cohorts, between 1970 and 2017. Participants in higher categories of BMI were more often of black race, more likely to have hypertension, diabetes, and albuminuria, and less likely to be current smokers. After long-term follow-up, individuals with a BMI > 30 kg/m^2^ belonging to the cohorts of the general population, showed a significantly higher risk for GFR decline, and a “J” shaped association between BMI and mortality, with the lowest risk for BMI of 25 kg/m^2^. In the cohorts with high cardiovascular risk and CKD, the risk association between high BMI and GFR decline was weaker than in the general population, with a “J” shaped association between BMI and death, with the lowest risk for BMI values between 25 and 30 kg/m^2^ (64).

A recent multicenter cross-sectional study involving 3,118 youth with overweight /obesity, and 286 healthy normal weight youth between 5 and 14 years of age. compared the prevalence of mildly reduced estimated GFR (eGFR > 60 and <90 mL/min/1.73 m^2^), and its association with cardiometabolic risk factors in overweight/ obese youth. eGFR was calculated using the updated bedside Schwartz equation and Full Age Spectrum (FAS) equation. The FAS equation identified a higher prevalence of youth with mildly reduced estimated GFR, compared to bedside Schwartz equation. Individuals with mildly reduced estimated glomerular filtration rate, according with both equations showed lower birth weight, younger age, higher BMI-SDS, non-high-density lipoprotein-cholesterol and serum uric acid, as compared to those with normal eGFR (65).

The association between BMI and risk of developing all-cause, diabetic, and non-diabetic end stage kidney disease (ESKD) was assessed in 1.2 million adolescents aged 17 years: over 25 years. This was a nation-wide population- based retrospective cohort study that linked medical data of 1,194,704 adolescents aged 17 years who had been examined for fitness for military service, between January 1, 1967, and December 31, 1997, to the Israeli ESRD registry. The overall incidence rate of CKD was 2.87 cases per 100,000 person- years. Compared to adolescents with normal weight, overweight and obese adolescents had an increased future risk for treated end- stage kidney disease (ESKD), with incidence rates of 6.08 and 13.40 cases per 100,000 person-years, respectively. Overweight and obesity were strongly associated with all-cause treated end- stage kidney disease [hazard ratio of 3.00 (95% CI, 2.50–3.60) and 6.89 (95% CI, 5.52–8.59), respectively], as well as, strong and independent risk factors for diabetic and non-diabetic kidney disease (66).

The effect of obesity on the kidney function could be time-dependent. In fact, duration of obesity negatively influences the eGFR in obese children (67) and in children with congenital solitary kidney (68). Moreover, the duration of obesity could increase the risk of development of kidney injury in adults with congenital solitary kidney (69). Obesity is a clinical condition associated with altered body composition. As discussed above, in this context, creatinine may not be the ideal biomarker for GFR measurement. This is especially true in obese children and adolescents, in whom besides the altered body composition; normalization of eGFR to 1.73 m^2^, introduces another bias. As BMI is strongly correlated with the body surface, the adjustment for body surface removes the effect of body weight on GFR, causing an underestimation of true GFR in individuals with a higher BMI, masking the installation of hyperfiltration (70). The challenges of measuring GFR in obese pediatric patients can be overcome either by replacing the real weight in the calculation of body surface by the ideal weight, calculated as,

**Ideal weight = BMI at 50th percentile (kg/m**^**2**^**) X Height** ^**2**^ **(m)** (71), or by using a height -independent creatinine -based equation for GFR estimation. Another possibility would the utilization of a sCysC -based equation for GFR estimation.

Weight-loss interventions slow or reverse early CKD progression, in adults and children (72–74). Pre- bariatric surgery data of 242 adolescents from the “Teen-Labs” study show microalbuminuria in 14%, macroalbuminuria in 3%, serum cystatin C-based eGFR <60 mL / min / 1.73 m^2^ in 3% and eGFR > 150 mL / min / 1.73 m^2^ in 7.1%. Lower eGFR was associated with higher values of BMI and/ or HOMA-IR. Three years post-surgery, patients with baseline serum cystatin C-based eGFR under 90 mL/min/1.73 m^2^, showed a significant improvement in mean eGFR from 76 to 102 mL/min/1.73 m^2^, while participants with albuminuria (albumin-to-creatinine ratio of 30 mg/g and more) at baseline presented a reduction in the geometric mean of ACR from 74 mg/g to 17 mg/g at 3 years follow-up. Patients with normal baseline renal function and no albuminuria at baseline remained stable throughout the study period. Among individuals with a BMI ≥ 40 kg/m^2^, increased BMI was associated with a significantly lower eGFR, while no association was observed in those with a BMI under 40 kg/m^2^. After adjusted analysis, post-surgical eGFR increased by 3.9 mL/min/1.73 m^2^ for each 10-unit loss of BMI (73). Studies in adults, however, show a consistent association between obesity and lower mortality in those with advanced CKD, especially among hemodialysis patients, suggesting that the reverse epidemiology of obesity is biologically reasonable (75).

### Final Commentaries

Obesity represents a major risk factor for the development of CKD. Sequential GFR determinations are the basis of renal function follow-up in affected individuals across all age groups. Obesity is also an example of a situation in which due to altered body composition, creatinine based GFR evaluation may lead to imprecise results. Additionally, some of the currently available creatinine GFR determination equations were developed in CKD patients' cohorts, which makes their utilization in individuals with normal GFR amenable to error (21, 28, 56).

Unfortunately, with the exception GFR determinations based on plasma disappearance of an intravenous injection of a low molecular weight substance that, on account of their precision, are routinely used for clinical research, there is no agreement on which is the best GFR determination equation to be used in children, adults or across the life span, as demonstrated by the multiple equations described above. This dilemma is expressed in a recent study, in obese children, in which the detection of changes in eGFR over time in response to therapeutic interventions were detectable only with the utilization of a height independent eGFR equation (74). Of note, another pediatric option of height independent GFR estimation is the equation proposed by the British Columbia Children's Hospital (BCCH; eGFR-BCCH), which in comparison to Potttel's equation might yield inferior results (76).

## Author Contributions

The author confirms being the sole contributor of this work and has approved it for publication.

## Conflict of Interest

The author declares that the research was conducted in the absence of any commercial or financial relationships that could be construed as a potential conflict of interest.

## Publisher's Note

All claims expressed in this article are solely those of the authors and do not necessarily represent those of their affiliated organizations, or those of the publisher, the editors and the reviewers. Any product that may be evaluated in this article, or claim that may be made by its manufacturer, is not guaranteed or endorsed by the publisher.

## Footnotes

^1^https://www.who.int/news-room/fact-sheets/detail/obesity-and-overweight (accessed Aug 22, 2021).
